# Management of liver trauma by laparoscopy using infrahepatic inferior vena cava partial clamping: A case report

**DOI:** 10.3389/fsurg.2022.1018953

**Published:** 2023-01-10

**Authors:** Dan Zhang, Ming-Da Tan, Ming-You Zheng, Huai-Zhi Wang, Lin-Kang Xiao

**Affiliations:** ^1^ Department of Radiology, Chongqing General Hospital, Chongqing, China; ^2^ Department of Hepatopancreatobiliary Surgery, Chongqing General Hospital, Chongqing, China

**Keywords:** infrahepatic inferior vena cava clamping, laparoscopy, liver trauma, hemodynamic instability, juxtahepatic venous injuries

## Abstract

Liver trauma with hemodynamic instability is extremely dangerous. Exploratory surgery after fluid resuscitation is a potentially effective method to save lives. Although there have been great advances in laparoscopic techniques for hepatectomy, laparoscopy is rarely used for liver trauma. According to our previous experience, laparoscopic infrahepatic inferior vena cava (IVC) clamping was a safe and effective technique to reduce central venous pressure (CVP) and control bleeding during hepatectomy. In this article, we described a case of grade V liver trauma that had been managed by an entirely laparoscopic approach using infrahepatic IVC partial clamping, outlining the technique of laparoscopy for liver trauma and the postoperative outcomes.

## Introduction

Liver trauma with hemodynamic instability is an extremely dangerous condition, usually with extensive liver parenchymal disruption or uncontrolled bleeding caused by juxtahepatic venous injuries, such as in the retrohepatic vena cava and central major hepatic veins (1). Emergency exploratory surgery after aggressive fluid resuscitation is a potentially effective way to save lives (2). Nowadays, laparoscopy has been maturely used for most abdominal surgical procedures, including hepatectomy. However, the application of laparoscopy for liver trauma has been slow to evolve partly due to the limitations of the laparoscopic technique and inherent factors of the trauma population, such as hemodynamic status, associated injuries, and anatomical liver injury grade (3). To control intraoperative bleeding, liver surgeons have evolved surgical techniques and designed Pringle maneuvers for hepatic vascular inﬂow occlusion and infrahepatic inferior vena cava (IVC) clamping for central venous pressure (CVP) reduction during parenchymal transection (4). These techniques may enable the application of laparoscopy for liver trauma. In this report, we describe an entirely laparoscopic approach for liver trauma using infrahepatic IVC partial clamping and outline the safety and feasibility of laparoscopy for liver trauma.

## Case description

**Figure 1:**
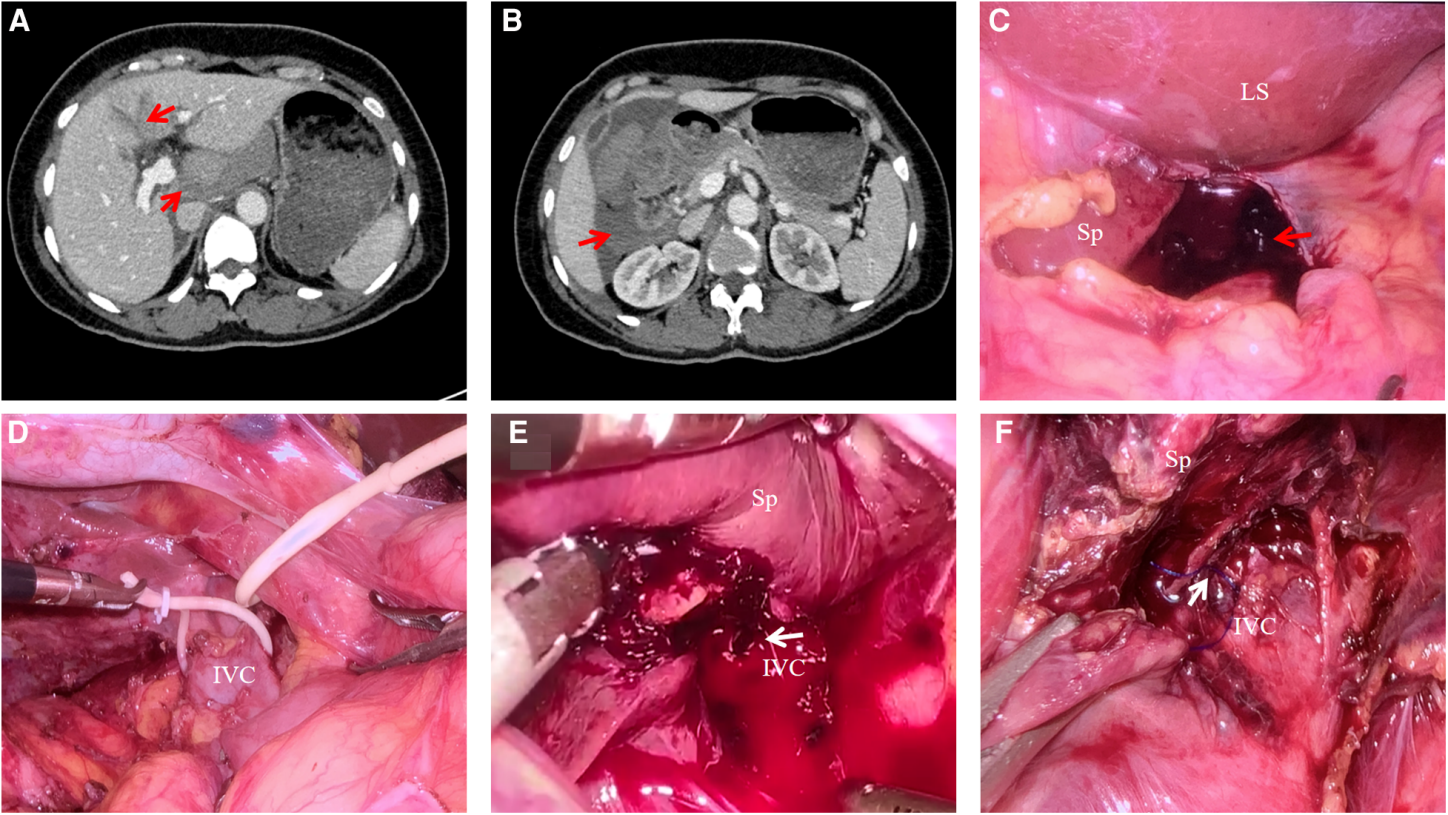
CT scan of liver trauma and procedure of laparoscopy by infrahepatic IVC clamping. (**A**) CT scan showing liver laceration involving segments I and IV; (**B**) CT scan showing extensive hemoperitoneum around the liver; (**C**) laparoscopy showing the perihepatic hemorrhage and blood clot; (**D**) infrahepatic IVC taped with a vessel loop; (**E**) laceration in the anterior wall of retrohepatic IVC was clearly exposed by laparoscopy without bleeding in the condition of the Pringle maneuver and infrahepatic IVC partial clamping; (**F**) laceration was safely sutured. Sp, spiegel lobe; Ls, left lateral section.

A 48-year-old female industrial worker without virus hepatitis and cirrhosis was admitted to the emergency room of Chongqing General Hospital with hemodynamic instability after falling from a height. The initial heart rate and blood pressure were recorded at 110 beats per minute and 80/65 mmHg, respectively. Abdominal pain and tenderness were obvious in the upper abdominal quadrants, and the focused abdominal sonography for trauma (FAST) returned a positive result. Aggressive fluid resuscitation with norepinephrine was conducted through the peripheral and central veins, increasing the blood pressure to normal, and then, a contrast-enhanced computed tomography (CT) scan was performed immediately under close monitoring. The CT scan showed hemoperitoneum, liver laceration involving segments I and IV, and rib fracture, without other concomitant organ injuries (Figures 1A,B). The presence of hypovolemic shocks, such as tachycardia and hypotension, was an ominous sign suggesting massive bleeding, so an emergency laparoscopy was performed immediately.

## Surgical procedure

The patient was positioned in the reversed Trendelenburg position with legs apart. Laparoscopy was performed with 5-mm trocars, and pneumoperitoneum pressure was maintained at 12 mmHg. The initial step of the operation was to occlude the hepatic vascular inﬂow by the Pringle maneuver. To reduce further bleeding, after suction of perihepatic hemorrhage, the location of bleeding was temporarily compressed with gauze and then the infrahepatic IVC was dissected below the hepatoduodenal ligament and taped with a vessel loop above the left renal vein (Figure 1D). The infrahepatic IVC clamping technique has been described previously (5). Briefly, the hemodynamic stability was guaranteed by gradual partial occlusion of the vessel loop and continuous monitoring of CVP and mean arterial pressure (MAP), with MAP not less than 65 mmHg. In addition, norepinephrine or a transfusion could be used to maintain hemodynamic stability if necessary. In the presence of hepatic vascular inﬂow occlusion and infrahepatic IVC partial clamping, a laceration in the anterior wall of retrohepatic IVC resulting from a hepatic short vein injury was detected after mobilization of the caudate lobe, and it was safely sutured by a 5-0 Prolene suture (Figures 1E,F). If the laceration was large, to reduce the risk of gas embolism or further bleeding, a laparoscopic bulldog clip could be applied to clamp part of the IVC wall before suturing if feasible. The infrahepatic IVC clamping loop was not loosened until the bleeding was completely controlled. The parenchymal laceration was coagulated with bipolar electrocoagulation for hemostasis. After complete hemostasis, the injury of other concomitant abdominal organs could be detected by laparoscopy if necessary, and abdominal drainage was placed in subphrenic space routinely.

## Results

The laparoscopy revealed a perihepatic hemorrhage of approximately 800 ml and parenchymal laceration of 3 cm and 4 cm depth in the Spiegel lobe and segment IV, respectively (Figures 1C,E). After hepatic vascular inﬂow occlusion and infrahepatic IVC partial clamping, a 2-mm laceration in the anterior wall of retrohepatic IVC resulting from a hepatic short vein injury was detected. According to the American Association for the Surgery of Trauma, the injury of the patient was classified as grade V liver trauma, and the patient was diagnosed with severe trauma with hemodynamic instability (6).

The patient was able to tolerate a partial clamping of the IVC. The time taken to establish infrahepatic IVC clamping with the laparoscopic approach was 12 min. The time of hepatic vascular inflow occlusion and the time of infrahepatic IVC partial clamping were 22 and 10 min, respectively. During hepatic vascular inflow occlusion and infrahepatic IVC partial clamping, the CVP and MAP were 0 cmH_2_O and 70 mmHg, which were decreased from 4 cmH_2_O and 80 mmHg, respectively. The blood loss during hemostasis was 100 ml. The intraoperative transfusion and crystalloid fluid infusion were 400 and 2,500 ml, respectively. The operation time was 1,50 min. The patient was discharged 7 days postoperation with an uneventful recovery, and there were no complications such as abscesses, bile leakage, hemobilia, biloma, or hepatic artery pseudoaneurysm during the 6-month follow-up.

## Discussion

Laparoscopy for liver trauma is considered a technically challenging procedure, especially in the case of severe hemorrhage (7). On the one hand, a large number of fluid infusions or blood products are required to ensure organ perfusion and maintain hemodynamic stability. On the other hand, the CVP needs to be reduced to facilitate hemostasis and the management of liver trauma. In this situation, laparoscopic infrahepatic IVC partial clamping may be an advisable technique to effectively reduce CVP without requiring fluid restriction (5).

In laparoscopy for liver trauma, once pneumoperitoneum was established, the Pringle maneuver should be performed immediately to reduce further bleeding rather than prioritizing the identification of the location of the injury. After initial control of bleeding by the Pringle maneuver, the infrahepatic IVC should be quickly dissected below the hepatoduodenal ligament and taped with a vessel loop above the left renal vein to control CVP and bleeding by temporarily clamping. With the Pringle maneuver and infrahepatic IVC partial clamping, a liver injury could be safely managed by suture, ligation or vascular repair, electrocoagulation, or even liver resection. It should be noted that CVP and mean arterial pressure (MAP) should be closely monitored during infrahepatic IVC partial clamping, and the MAP should not be less than 65 mmHg, which could be achieved by norepinephrine or a transfusion if necessary. In addition, if the liver injury was serious or hemodynamic status was difficult to maintain stability, laparoscopy was a dangerous procedure, and timely conversion to open laparotomy was required.

In this patient, the hemodynamic status was quickly restored to stability after aggressive fluid resuscitation with norepinephrine, and was in a stable condition for the Pringle maneuver and infrahepatic IVC partial clamping. The establishment of infrahepatic IVC clamping using the laparoscopic approach was safe and feasible without a long time consumption or related injury. Parenchyma disruption and injury of a hepatic short vein, resulting in massive bleeding and hemodynamic instability, were detected by a laparoscopy. During the application of the Pringle maneuver and infrahepatic IVC partial clamping, the laceration in the anterior wall of the retrohepatic IVC was safely sutured and the parenchymal laceration was coagulated with bipolar electrocoagulation. There were no postoperative complications related to infrahepatic IVC partial clamping, and the patient recovered rapidly.

A laparoscopy for liver trauma has several advantages. The wide field of vision and magnification could facilitate the detection of other associated injuries, and pneumoperitoneum pressure may be beneficial in reducing bleeding. Moreover, laparoscopy is minimally invasive and is easy to recover from after surgery. However, laparoscopy as an initial approach for liver trauma is not recommended. Currently, the majority of patients with blunt liver trauma, especially children, were treated with a nonoperative management strategy, while those with hemodynamic instability and nonresponsiveness should undergo an operation (8, 9). Other indications for an operation were some complications associated with nonoperative management strategies, such as hemorrhage, bile peritonitis, abdominal compartment syndrome, and liver compression (10). In this situation, a laparoscopy was considered the optimal management approach to minimize the invasiveness of surgical intervention and to tailor the procedure to the lesion (11).

In fact, laparoscopy for liver trauma is fraught with risks and challenges. It is essential to consider these factors when choosing laparoscopy for liver trauma including hemodynamic status, associated injuries, anatomical liver injury grade, and surgeon’s experience. Usually, hepatectomy is not recommended as a priority for liver trauma; stopping bleeding and saving a life should be the primary task. Meanwhile, the damage control procedures (DCS) should be strictly followed, which usually require exploratory laparotomy for severe liver trauma. Laparoscopy might only be considered in patients who could be stabilized, are eligible to undergo a previous CT scan to reveal the injury pattern, and present with an abdominal monoinjury. Thus, a laparoscopy may have no value in a true DCS setting in the case of severe liver trauma or concomitant other organ injuries. According to our experience, for a few liver traumas involving vascular injury with hemodynamic stability after aggressive fluid resuscitation, the laparoscopic approach using infrahepatic IVC partial clamping may be attempted by an experienced team, but for the major juxtahepatic and central hepatic venous injuries, the laparoscopy should be treated with caution.

This case may suggest that an entirely laparoscopic approach using infrahepatic IVC partial clamping for the treatment of liver trauma is feasible in an experienced center for well-selected patients. However, owing to the complexity of liver trauma and the risks associated with laparoscopy, it should be considered an exceptional option that is not routinely applied even in hemodynamically stable patients.

## Data Availability

The raw data supporting the conclusions of this article will be made available by the authors without undue reservation.
